# Unlocking the *Ilex guayusa* Potential: Volatile Composition, Antioxidant, Antidiabetic, and Hemolytic Activities, with In Silico Molecular Docking and ADMET Analysis of Hydroethanolic Extracts

**DOI:** 10.3390/molecules30193885

**Published:** 2025-09-25

**Authors:** Nina Espinosa de los Monteros-Silva, Karla Martínez-Palacios, Anggie M. Jiménez, Melanie Ochoa-Ocampo, Thomas Garzón, Tamara Carrillo-Vásconez, Matteo Radice, Enith Vanessa Yánez, Julio Rea-Martínez, Zulay Niño-Ruiz, Karel Dieguez-Santana, Noroska G. S. Mogollón

**Affiliations:** 1 Laboratorio de Biología Molecular y Bioquímica, Universidad Regional Amazónica Ikiam, Km7 Via Muyuna, Tena 150101, Ecuador; nina.espinosadelosmonteros@ikiam.edu.ec; 2 Biomolecules Discovery Group, Universidad Regional Amazónica Ikiam, Km7 Via Muyuna, Tena 150101, Ecuador; karla.martinez@est.ikiam.edu.ec (K.M.-P.); anggie.medina@est.ikiam.edu.ec (A.M.J.); thomas.garzon@ikiam.edu.ec (T.G.); tamara.carrillo@est.ikiam.edu.ec (T.C.-V.); julio.rea@ikiam.edu.ec (J.R.-M.); karel.dieguez@ikiam.edu.ec (K.D.-S.); 3 Laboratorio de Productos Naturales, Universidad Regional Amazónica Ikiam, Km7 Via Muyuna, Tena 150101, Ecuador; melanie.ochoa@ikiam.edu.ec; 4 Facultad de Ciencias de la Tierra, Universidad Estatal Amazónica, Puyo 160150, Ecuador; mradice@uea.edu.ec; 5 Grupo Traslacional en Plantas, Universidad Regional Amazónica Ikiam, Km7 Via Muyuna, Tena 150101, Ecuador; vanessa.yanez@ikiam.edu.ec; 6 Biomass to Resources Group, Universidad Regional Amazónica Ikiam, Km 7 Vía Muyuna, Tena 150101, Ecuador; zulay.nino@ikiam.edu.ec

**Keywords:** plants, biological activity, amazon, secondary metabolites, GC-MS

## Abstract

This work studies the underexplored potential of Ilex guayusa and demonstrates the influence of geographical (locations: A, B, C) and ontogenetic (young: 0; old: 2) factors on its biochemical profile. The total phenolic content (TPC) was consistently higher than the total flavonoid content (TFC) in all samples, with the highest values for site B: B2 for TPC (77.91 μg GAE/mg extract) and B0 for TFC (6.77 μg QE/mg extract). GC–MS identified 29 metabolites, and clustering analysis grouped samples B and C as rich in phenols and flavonoids, while site A was richer in alcohols, aldehydes, and hydrocarbons. Antioxidant potential was demonstrated, with B2 being the most active in ABTS (TEAC value of 0.3885 (mg/g dw)), whereas A2 and C2 showed the strongest activity in DPPH (0.0968 and 0.1850 (mg/g dw), respectively). No sample exhibited hemolysis and α-amylase inhibition; however, α-glucosidase inhibition was observed with the best activity for B0 (IC_50_ = 68.05 µg/mL). Molecular docking, ADME, and correlation analyses indicated that B0 had the highest TFC, DPPH, and α-glucosidase inhibition values, while B2 showed the highest TPC and ABTS activity. Overall, the promising antioxidant and hypoglycemic activity combined with low toxicity highlights and expands the therapeutic and applicative potential of the species.

## 1. Introduction

The Amazon is home to a great variety of plant and animal species and is also the site where about ten Indigenous nationalities have settled [1]. For years, this dynamic interaction between nature and humans has established an empirical system of natural resource management and the generation of ancestral knowledge based on observation [2]. Within Ecuador’s extensive biodiversity, plants provide important ecosystem services, including food, medicine, and fuel, among others [3]. This region has one of the highest records of medicinal plant use in South America as a treatment for diseases [4]. Among these species, we find *Ilex guayusa*, currently famous for its energizing properties and for which its ethnopharmacological use has been reported to treat gastrointestinal problems, body aches, colds, and infertility in women [5,6,7].

*I. guayusa* is a shrub belonging to the Aquifoliaceae family. It can reach a height of between 6 and 10 m and is native to the Amazon region. The species is distributed in the Amazon foothills and along the slopes of the Andes and is found in Bolivia, Ecuador, Colombia and Peru [8]. Ethnobotanical information reports the use of guayusa infusion to increase fertility and libido, to treat diabetes, certain sexually transmitted diseases, and muscle pain [9]. The first preliminary study on the anti-glycemic effects of *I. guayusa* extracts dates to 1989 [10]. The research mentioned the ability of an aqueous extract of guayusa to delay the development of hyperglycemia in animal models. Although further research is needed, the study by Swantson and Flat highlighted an initial correlation between ethnomedical information and experimental data [10].

All the above-mentioned pharmaceutical properties are closely related to their diversity of chemical components, usually divided into three types: terpenes (or terpenoids), nitrogen-containing compounds (such as alkaloids, cyanogenic glycosides, glucosinolates, and nonprotein amino acids), and phenolic compounds (such as flavonoids, isoflavonoids, anthocyanins, lignins, and tannins) [11]. They can vary and accumulate in response to different stress factors, both abiotic (shade, humidity, injuries, soil conditions, age) and biotic (bacteria, fungi, nematodes), thus contributing to the adaptation and permanence of the species in their ecosystem [12,13,14]. Within these factors, the geographical conditions in which crops develop, such as altitude, significantly influence their growth, survival, and metabolism [15]. Additionally, factors such as plant age can affect their chemical composition due to their accumulation at specific stages of the plant development [16,17]. However, there are not too many studies analyzing the best conditions of samples needed to improve the potential of plants. Therefore, it could be a good way to lead the discovery of new molecules and alternatives to face problems in the medical field, such as searching for biological resources with antioxidant, antidiabetic, and neuroprotective activities.

In this context, plant extracts with antioxidant activity are valuable with a pharmaceutical aim, because they can protect the organisms against free radicals causing damage [18,19]. Oxidative stress reduction is closely related to the fight against some cardiovascular, inflammatory, cancer, anemia, and aging problems [20,21]. Moreover, natural antioxidants have been lately highlighted because several represent lower costs and promising results. In particular, medicinal plants have been reported to have great antioxidant potential, but they still represent a significant group of unexplored species in this field. In addition, potential antidiabetic properties of plants are relevant as a strategy to face Type 2 diabetes mellitus (T2DM), which is a progressive metabolic disorder characterized by insulin resistance and impaired insulin secretion, leading to chronic hyperglycemia and long-term complications affecting the cardiovascular, renal, and nervous systems [22]. Current strategies include lifestyle modifications, oral hypoglycemic agents, and in some cases, insulin therapy [23]. As the global prevalence of T2DM continues to rise, searching for new strategies to face it remains a critical public health priority, where plant extracts can be a good option [24].

In addition to evaluating the potential biological activities of plants, it is essential to assess their toxicity at various levels, including cytotoxic, acute, subchronic, and chronic effects [25,26,27]. Despite its significance, toxicological research on plant species remains relatively underexplored compared to the extensive focus placed on their pharmacological or industrial applications [28]. This gap in knowledge poses a major limitation when considering the transition from preliminary studies to the real-world application of these species in areas such as medicine, food, or cosmetics [25,28,29]. Comprehensive toxicity profiling not only ensures safety for human and environmental health, but also supports regulatory approval processes and the sustainable exploitation of natural resources [29]. Therefore, integrating toxicological evaluation into early stages of research is fundamental to promoting responsible and evidence-based use of plant-derived compounds.

Despite the reported potential of plant extracts, the aforementioned properties of guayusa and the factors that can influence them, studies have mostly focused on analyzing its energizing capacity, leaving aside the opportunity to promote an integral and better use of the species. In this context, the present study aims to evaluate the hypoglycemic activity and chemical composition of *I. guayusa* under various geographical conditions and plant ages. It aims to promote the development of new therapeutic strategies based on natural compounds, aligning with current medical needs. In addition, it will contribute to the economic dynamism of the communities that commercialize this species, giving added value to this biological resource of cultural importance in the Ecuadorian Amazon Region.

## 2. Results

### 2.1. Total Phenolics and Flavonoids Content

Quantitative analysis revealed significant variations in total phenolic and flavonoid contents among *Ilex guayusa* leaves collected from different sites and at different ages (Table 1). Total phenolic content ranged from 34.58 ± 0.25 μg GAE/mg extract in A2 to 77.91 ± 0.16 μg GAE/mg extract in B2. In contrast, total flavonoid content varied from 4.27 ± 0.12 μg QE/mg extract in C2 to 6.77 ± 0.07 μg QE/mg extract in B0. The methodologies employed for these analyses, along with data processing steps, are detailed in the Github repository (https://github.com/SaborCanela/Unlocking-the-Ilex-guayusa-Potential-Volatile-Composition-Antioxidant-Antidiabetic, accessed on 10 August 2025).

### 2.2. Antioxidant Assay

Antioxidant activity, assessed through the ABTS and DPPH methods, is summarized in Table 2. Values are expressed as Trolox equivalent antioxidant capacity (mg Trolox equivalents/g dry weight) and reported as mean ± SD from triplicate determinations.

### 2.3. Antidiabetic Potential of the Extracts

#### 2.3.1. Effect of the Extracts on α-Amylase Inhibition

**Figure 1:**
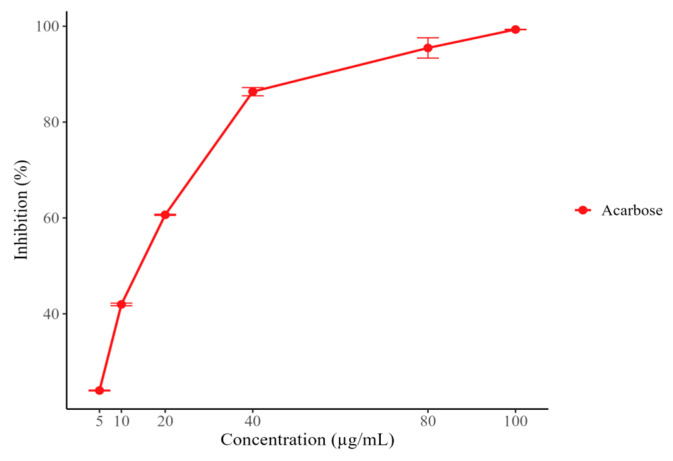
α-amylase inhibition percentage (%) of Acarbose.

None of the samples evaluated exhibited activity at the tested concentrations. In contrast, acarbose showed marked activity, reaching 99.32% inhibition at the highest concentration tested (100 µg/mL), with an IC_50_ value of 14.30 ± 0.55 µg/mL (Figure 1).

#### 2.3.2. Effect of the Extracts on α-Glucosidase Inhibition

**Figure 2:**
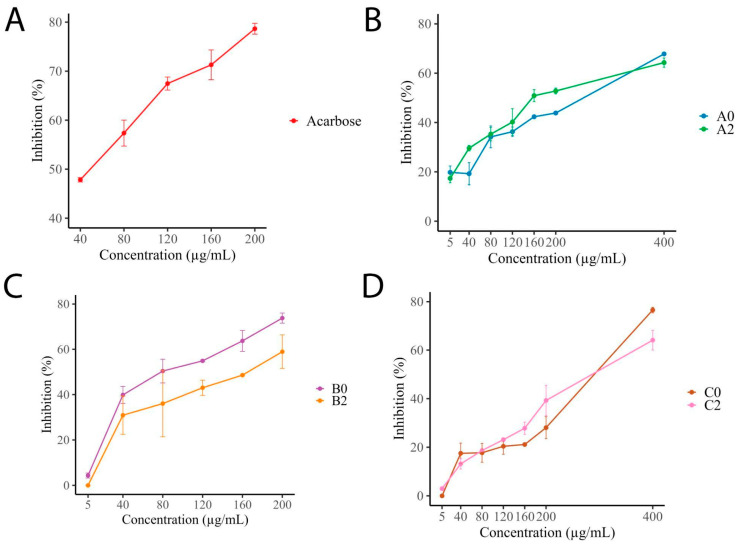
Inhibition percentage (%) of α-glucosidase by *I. guayusa* hydroethanolic extracts. (**A**) Acarbose. (**B**) Alto Talag. (**C**) Alto Pano. (**D**) Alto Tena. 0: Young leaves. 2: Old leaves.

The enzyme inhibition results, summarized in Figure 2, indicate that the six *I. guayusa* extracts exhibited inhibitory activity against α-glucosidase. The positive control, acarbose, achieved 78% inhibition at 200 μg/mL. Samples of Alto Talag (A) showed that both young (A0) and old leaves (A2) reached 63% and 67% inhibition at 400 μg/mL, respectively, with minimal differences between age groups. B samples displayed notable age-related differences in their inhibition profiles. B0 showed 70% inhibition at 200 μg/mL, while B2 reached only 50% inhibition at the same concentration. Compared to the positive control, B0 displayed the highest activity among all tested samples at equivalent concentrations. C samples showed lower inhibition percentages up to 200 μg/mL, then increased their activity at 400 μg/mL, reaching 70% inhibition (C0) and 60% (C2).

IC_50_ values revealed differences in inhibitory potency among the extracts (Table 3). Acarbose exhibited the lowest IC_50_ value (48.79 ± 0.80 μg/mL), followed by B0 (68.05 ± 5.60 μg/mL), which demonstrated the highest activity among plant extracts. A2 and B2 showed moderate activity, with IC_50_ values of 158.92 ± 11.30 μg/mL and 172.85 ± 8.06 μg/mL, respectively. In contrast, A0, C0, and C2 exhibited IC_50_ values higher than 200 μg/mL, indicating lower inhibitory potency. Additionally, no significant differences were found between C0 and C2.

### 2.4. GC-MS-Based Metabolite Identification

A gas chromatography–mass spectrometry (GC-MS) analysis was carried out to explore the profile of volatile and semi-volatile metabolites in the ethanolic extracts of *I. guayusa*. In total, 29 compounds were detected. Of these, two were unequivocally identified as Level 1 compounds by direct comparison with authentic reference standards. Twelve were annotated as Level 2 based on high spectral similarity with entries from the NIST 20 EI library, MS-DIAL database, and the GNPS platform (ID = 8df6fcabd7f74f938bddec3e13c0d15d), combined with retention index matching using the Van den Dool and Kratz method. The remaining fifteen compounds were assigned to Level 3, based on interpretation of their mass spectral fragmentation patterns and molecular weight (Table 4).

**Figure 3:**
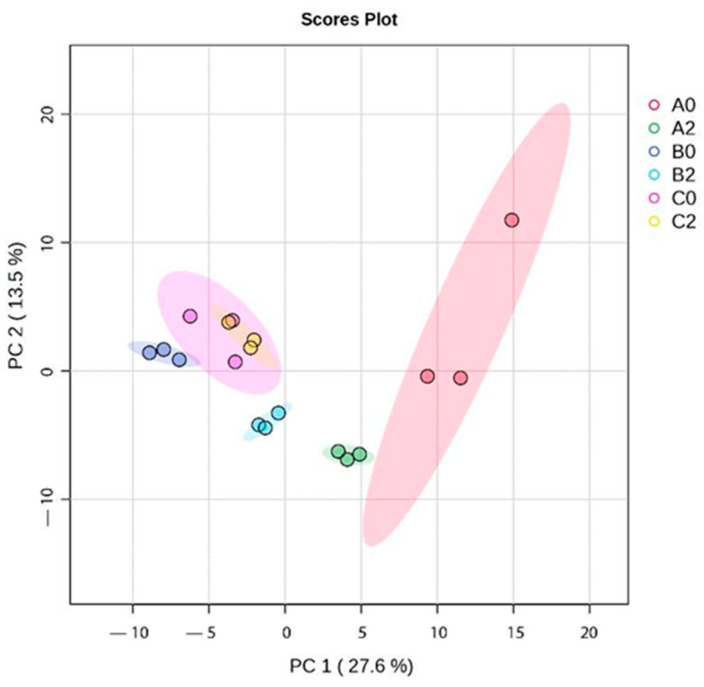
Principal component analysis (PCA) of *I.guayusa* hydroethanolic extracts.

Additionally, the Principal Component Analysis (PCA) revealed distinct clustering patterns among *I. guayusa* samples based on locality and leaf maturity stage (Figure 3). PC1 accounted for 27.6% of the total variance and PC2 for 13.5%. Overall, two main clusters were observed: all samples of Alto Pano (B) and Alto Tena (C) grouped in the negative PC1 region, while Alto Talag (A) samples clustered in the positive PC1 region. Within A, leaf maturity also influenced separation: A0 were positioned toward the positive PC1 axis but remained slightly apart from A2. In Alto Pano (B), B0 clustered tightly in the lower left quadrant along the negative PC1, whereas B2 were also in the negative PC1 region but shifted slightly toward the center, suggesting maturity-related metabolomic differences. In Alto Tena (C), C0 and C2 clustered closely in the upper left quadrant along negative PC1, showing minimal separation by maturity stage and indicating smaller metabolomic differences compared to Alto Talag (A) and Alto Pano (B).

**Figure 4:**
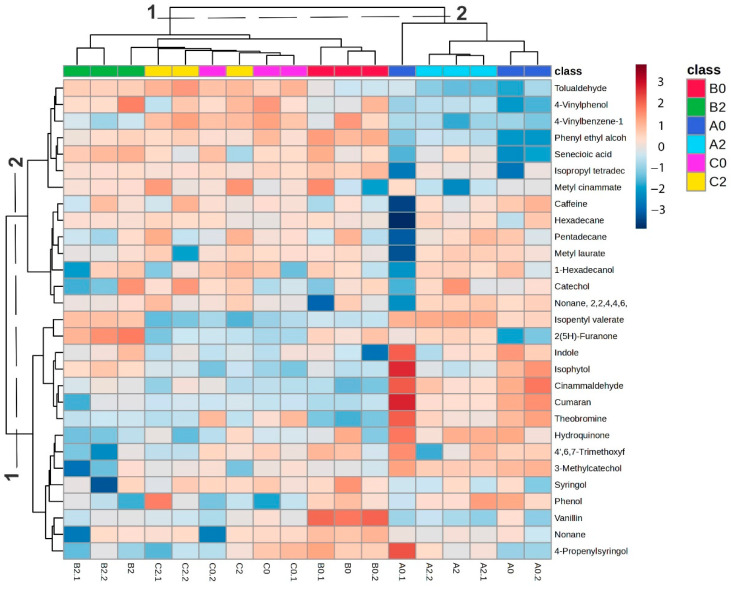
Heatmap and HCA; cold colors (blue scale) represent a low metabolite abundance, while warmer colors (red scale) indicate a higher abundance.

The variability of components in the samples was assessed using heatmap visualization combined with hierarchical cluster analysis (HCA) (Figure 4). The clustering pattern closely reflected the results obtained from PCA. In the upper heatmap cluster (Group 1), which included samples from B and C, higher relative abundances of benzenoids, lipids, and organoheterocyclic compounds were observed. Specifically, tolualdehyde, 4-vinylphenol, and 4-vinylbenzene-1-ol were particularly enriched in C2, whereas their levels were generally lower and more variable in B0 and B2. In contrast, phenethyl alcohol, senecioic acid, isopropyl tetradecyl ether, vanillin, syringol, and methyl cinnamate were most abundant in B0, present at moderate levels in B2, and displayed greater variability across C samples. The heatmap cluster, predominantly composed of Alto Talag (A) samples, revealed clear differences between leaf ages. Methyl laurate, 1-hexadecanol, catechol, isopentyl valerate, and 2(5H)-furanone were more abundant in A2 leaves, whereas indole, isophytol, cinnamaldehyde, coumaran, theobromine, 4′,6,7-trimethoxyflavonol, and 3-methylcatechol predominated in A0. These observations indicate a pronounced variation in A’s chemical profile according to leaf age, with specific benzenoids, lipids, and organoheterocyclic compounds accumulating preferentially at distinct developmental stages.

### 2.5. Correlation Between Metabolite Profile, Antioxidant Activity, and α-Glucosidase Inhibition

**Figure 5:**
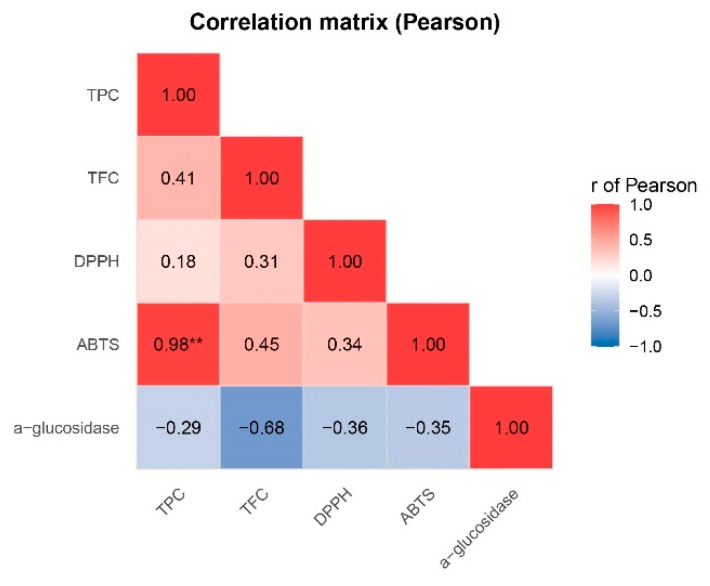
Pearson correlation between TPC, TFC, ABTS, DPPH, and α-glucosidase inhibition. ** = correlation is statistically significant at *p* < 0.001.

The Pearson correlation analysis (Figure 5) revealed distinct relationships among the evaluated bioactive parameters. As expected, TPC showed a very strong positive correlation with ABTS activity (r = 0.98, *p* < 0.05). On the other hand, moderate positive correlations were also observed between TPC and TFC (r = 0.41) and between TFC and ABTS (r = 0.45), suggesting a synergistic contribution of flavonoids to the overall antioxidant potential. In contrast, DPPH exhibited weaker correlations with the other antioxidant parameters, which may reflect differences in the radical systems or sensitivity of this assay compared to ABTS.

However, α-glucosidase inhibition displayed inverse correlations with all antioxidant-related parameters, particularly with TFC (r = –0.68). Since α-glucosidase values are expressed as percentage inhibition (IC_50_), stronger activity corresponds to more negative correlations (blue color in the heatmap).

**Figure 6:**
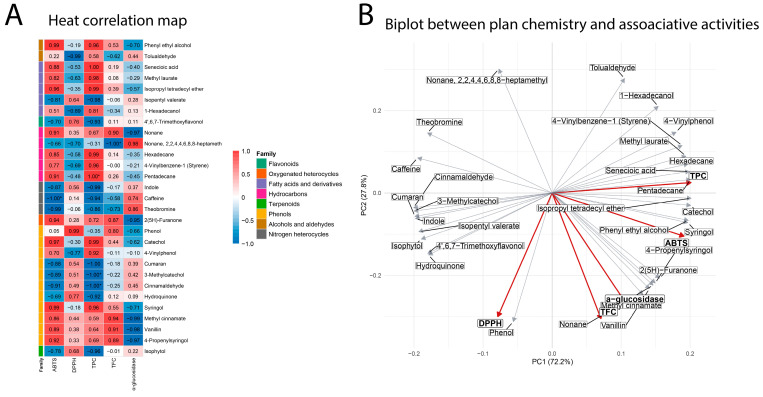
(**A**) Heat correlation map. (**B**) Biplot between *I. guayusa* profile and associated activities. * = correlation is statistically significant at *p* < 0.05. Red labels represent biological assays or activities, while black labels represent associated metabolites or chemical variables.

Figure 6 illustrates the relationship between the identified metabolites and their associated bioactivities. The heatmap revealed distinct assay–metabolite patterns, with correlations ranging from −1.0 to 0.99 across chemical families (Figure 6A). Strong α-glucosidase inhibition, as well as high correlations with TFC and ABTS and lower in DPPH, were associated with phenolic compounds, including methyl cinnamate, vanillin, 4-propenylsyringol, syringol, phenol, and catechol. These components cluster in the same PCA quadrant as α-glucosidase inhibition, ABTS, and TFC (Figure 6B), highlighting the correlations among these assays.

### 2.6. Hemolytic Activity of the Extracts

**Figure 7:**
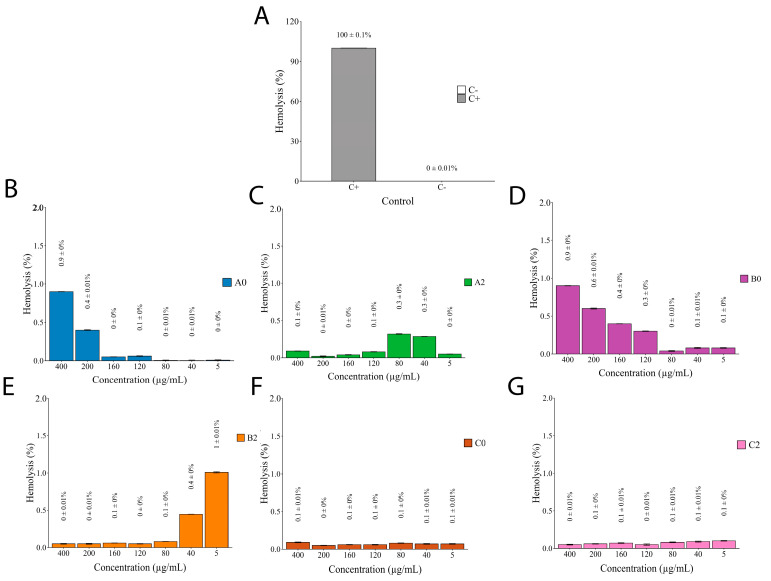
Hemolytic activity of *I.guayusa* extracts. (**A**) C+: Triton 2.5×. C-: PBS 1X. (**B**) A0 (Alto Talag—Young leaves) (**C**) A2 (Alto Talag—Old leaves). (**D**) B0 (Alto Pano—Young leaves). (**E**) B2 (Alto Pano—Old leaves). (**F**) C0 (Alto Tena—Young leaves). (**G**) C2 (Alto Tena—Old Leaves).

The hemolytic activity of *I. guayusa* extracts was evaluated in young and old leaves collected from different locations (Figure 7).

### 2.7. Docking Molecular

**Figure 8:**
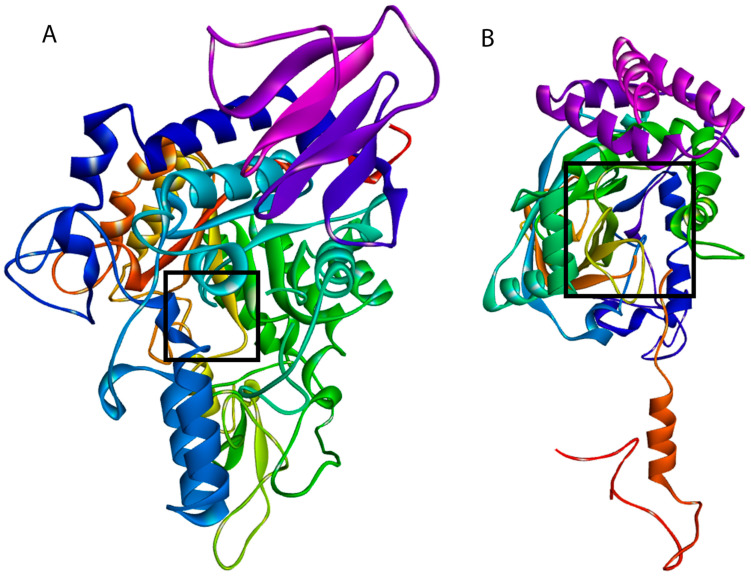
Schematic representation of enzymes. (**A**) α-glucosidase (3A4A), and (**B**) catalase (2CAG).

The metabolites of *I. guayusa* exhibited varying affinities toward α-glucosidase (3A4A) and catalase (2CAG) (Table 5). To provide structural context for the docking studies, Figure 8 illustrates the overall 3D structures of the target enzymes, α-glucosidase (3A4A) and catalase (2CAG), highlighting their complex topology and the location of the active sites where the interactions described below take place.

4′,6,7-trimethoxyflavonol was the compound with the highest affinity, with ΔG values of –8.3 kcal/mol (3A4A α-glucosidase) and –9.4 kcal/mol (2CAG catalase). This affinity was driven by specific interactions: in α-glucosidase, it established H-bonds with Arg315 and Asp242, along with T-shaped π–π stacking with Tyr158; in catalase, H-bonds with Arg51/Arg344 and π–π stacking with Tyr337 stood out.

**Figure 9:**
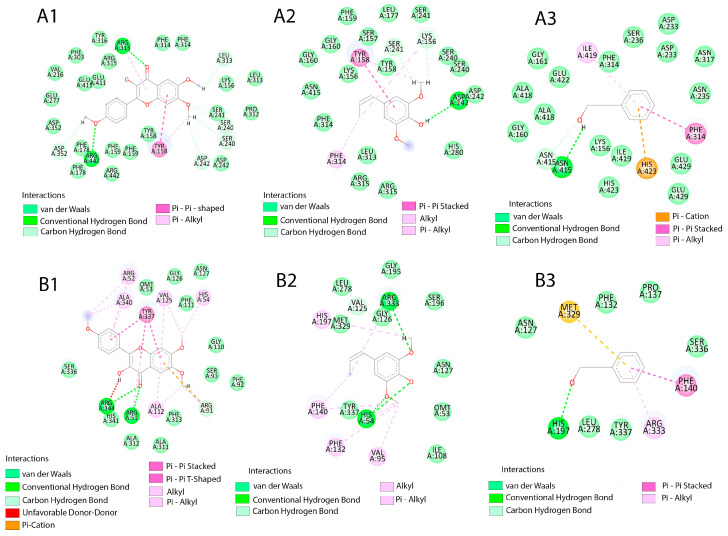
Schematic representation of the molecular interactions of the ligands, A: α-glucosidase (3A4A), and B: catalase (2CAG). 1: 4′,6,7-Trimethoxyflavonol, 2: 4-Propenylsyringol. 3: Vanillin.

Metabolites with intermediate affinity included 4-propenylsyringol (7), which showed ΔG of, −5.5 (3A4A), and −7.0 kcal/mol (2CAG). In catalase, this compound established key interactions such as π-alkyl with His197 and H-bonds with Arg333. Vanillin (5) (−5.9 kcal/mol in 3A4A) and syringol (8) (−5.8 kcal/mol in 3A4A) presented π-cation networks with His423, although vanillin exhibited structural impediment due to an unfavorable donor–donor interaction with Asn317. Among the low-affinity ligands, phenol (6), catechol (4), and Senecioic acid (2) showed energies ≥ −5.6 kcal/mol; phenol established π-sulfur contacts with Met329 in catalase. The specific molecular interactions for the top ligands are schematically summarized in Figure 9.

The docking protocol was first validated by redocking the positive control, acarbose, into the active site of the 3A4A protein. Acarbose, a clinical α-glucosidase inhibitor, produced a binding affinity (ΔG) of −9.0 kcal/mol, serving as a rigorous benchmark for high-affinity binding (See Figure S1). Subsequent screening of *I. guayusa* metabolites identified 4′,6,7-trimethoxyflavonol as the top candidate, with a ΔG of −8.3 kcal/mol. This computationally derived affinity, which is remarkably close to that of the established inhibitor acarbose, strongly supports the potent in vitro inhibitory activity (IC_50_ = 68.05 µg/mL) measured for the B0 extract where this compound was enriched.

A positive correlation was identified between the complexity of non-covalent interactions and affinity. Compounds with aromatic substituents (methoxy, propenyl) generated networks of H-bonds, π-stacking, and simultaneous hydrophobic contacts, anchoring themselves in catalytic pockets. In contrast, small ligands without these motifs showed weak and peripheral bonds, demonstrating that structural multivalency is critical for enzyme inhibition.

The pharmacokinetic parameters (ADME) of the nine metabolites of *Ilex guayusa* were evaluated using in silico tools (SwissADME, ADMETlab 3.0, pkCSM) (Table 6). All compounds exhibited high gastrointestinal absorption (>77%), meeting the optimal criterion for oral drugs. Permeability in Caco-2 cells (an intestinal barrier model) was favorable for all compounds (log Papp: 1.087–1.613 × 10^−6^ cm/s), with Comp6 (1.613) showing the highest value. Water solubility ranged from “very soluble” (Comp1, Comp2, Comp4, Comp5, Comp6, Comp8) to “moderately soluble” (Comp3, Comp9), with Comp3 displaying the lowest solubility (LogS = −5.096 mol/L). No compound violated Lipinski’s Rules (0 violations), confirming their drug-likeness. However, Comp3 and Comp9 presented lead-likeness problems (3 and 0 violations, respectively), associated with their moderate molecular weight (214.34 and 328.32 g/mol) and number of molecular rotations (nRB = 11 and 4).

Regarding distribution, all compounds showed low blood–brain barrier permeability (log BB < –0.089) and minimal CNS penetration (log PS < –1.661), suggesting a low risk of neurotoxicity. Comp3 had the highest plasma free fraction (0.23), whereas Comp9 had the lowest (0.14), indicating strong protein binding. In terms of metabolism, Comp9 was a substrate of CYP3A4 and an inhibitor of CYP1A2, CYP2C19, and CYP3A4, indicating potential drug interactions. The other compounds did not inhibit relevant CYP450 isoenzymes. For excretion, Comp3 displayed the highest renal clearance (logCL = 1.724 mL/min/kg), while Comp4, Comp6, and Comp8 had the lowest (<0.25 mL/min/kg). Only Comp9 was a substrate for OCT2, implying possible renal accumulation. While these computational ADMET estimates provide critical early insights for candidate prioritization, they are predictive models that require empirical confirmation. Future work will therefore focus on experimental validation through established in vitro methods, such as Caco-2 and CYP450 assays, to substantiate the predictions prior to any clinical application.

The toxicological profile of the nine compounds of *I. guayusa* extracts, evaluated using ProTox-3.0, revealed that Comp1, Comp2, Comp3, Comp5, Comp6, and Comp8 exhibited no hepatotoxic, carcinogenic, mutagenic, or cytotoxic activity, with probabilities scores (Pb) generally below 0.8, supporting their potential safety (Table 7). Two compounds, however, merit closer attention. Catechol (Comp4) showed a clear carcinogenic potential (Pb = 0.84), while phenol (Comp6) exhibited high acute toxicity (LD_50_ = 270 mg/kg, Class 3). While these predictions reflect isolated compound properties, they cannot be directly extrapolated to the whole extract formulation. Both compounds were detected in metabolomic profiling (Table 4), though their relative abundance remained low (catechol: 0.01–1.77%; phenol: 0.03–0.07%). The in vitro hemolysis assay (Figure 7) provides more relevant safety evidence, showing negligible hemolytic activity (<1%) across all samples—including the most active α-glucosidase inhibitory fraction, B0 (IC_50_ ≈ 68 µg/mL). This suggests that the limited abundance of these compounds, combined with potential matrix interactions within the extract, effectively mitigates any practical risk. Nevertheless, since only relative abundances were obtained in this study, absolute quantification of phenol and catechol will be required to more rigorously evaluate cumulative or chronic exposure scenarios, particularly in the context of nutraceutical applications. Such targeted quantification, together with repeated-dose toxicity assays, would provide a more robust framework to assess potential long-term risks.

## 3. Discussion

Guayusa demonstrated its notably higher quantity of phenolic than flavonoids content independently of age and cultivation site (Table 1). Despite no pattern being observed for the age condition, the highest content of phenolic compounds was found in B2. It could be related to the older leaves undergoing oxidative stress caused by chlorophyll degradation, light exposure, or changing environmental conditions [30]. In response, they synthesize more phenolic compounds as an antioxidant defense mechanism [30]. Similarly, Alto Pano (B) exhibited the highest flavonoid content, with the highest content in B0. This ontogenetic variation suggests a developmental regulation of secondary metabolites, likely driven by differential gene expression and the physiological requirements associated with distinct growth stages. Comparable trends have been reported in other species, such as *Cleome gynandra* where younger vegetative tissues showed markedly higher flavonoid levels, while phenolics predominantly accumulated in reproductive organs such as flowers and siliques at maturity [31]. Likewise, in wampee (*Clausena lansium*), total flavonoid content increased significantly in older leaves, while phenolic levels exhibited the opposite trend [32]. These findings underscore the importance of harvest timing for maximizing specific bioactive compounds and support the strategic use of plant developmental stages to optimize phytochemical yields in natural product research.

From them, the relevant role of flavonoids and polyphenols in biological activity is particularly well known. In plants, polyphenols are central to structural stability, lignification, and pathogen resistance [33]. Conversely, flavonoids function as UV filters and ROS scavengers and play an important role in defense responses against environmental stress. Chemical structure allows both of them metabolite class to donate electrons and neutralize reactive oxygen species (ROS), thereby reducing oxidative stress, which is a key factor not only as antioxidant properties but in the development of metabolic diseases [34].

In this context, significant differences were revealed between DPPH and ABTS assays for each sample (Table 2), highlighting B2 as the most active against ABTS radicals. Meanwhile, in the DPPH assay, A0 and B0 exhibited the strongest radical-scavenging activity, with no significant differences between them. These discrepancies can be attributed to the intrinsic nature of the methods: while DPPH is more selective toward lipophilic antioxidants, ABTS can react with both hydrophilic and lipophilic compounds, thereby providing a broader spectrum of radical-scavenging capacity [35]. Consequently, the higher ABTS activity observed in old leaves may be associated with their increased accumulation of total phenolics, whereas the stronger DPPH response in young leaves could be explained by a greater contribution of glycosylate flavonoids and other relatively lipophilic metabolites.

Moreover, for antidiabetic activity phenolic and flavonoid compounds have been documented to have the capacity to modulate oxidative stress, regulate inflammatory processes, and activate protective pathways in pancreatic beta cells [36]. These activities are relevant in the context of type 2 diabetes mellitus, because they contribute to mitigating the complications derived from this disease, especially those associated with damage induced by reactive oxygen species [37]. Thus, the enzymatic assays demonstrated inhibition of α-glucosidase, whereas no inhibitory effect was observed against α-amylase (Figure 1 and Figure 2). This pattern is commonly reported for plant extracts and is often attributed to the greater accessibility of the α-glucosidase active site compared to that of α-amylase [38,39]. In this study, the highest α-glucosidase inhibition was observed in B0, which also exhibited a higher flavonoid content and stronger DPPH radical-scavenging activity. Such a selective inhibition profile is particularly relevant from a pharmacological perspective, since molecules with higher affinity for α-glucosidase are preferred in the management of type 2 diabetes due to their ability to gradually modulate postprandial glucose release [40,41]. Moreover, targeting α-glucosidase while sparing α-amylase reduces the risk of adverse effects such as diarrhea and excessive intestinal fermentation, which are often associated with strong α-amylase inhibition [42,43,44].

In order to characterize the volatile profile, a GC-MS analysis was carried out, which revealed a total of 29 metabolites, of which several have reported biological activity (Table 4). Regarding the location, PCA revealed a separation between Alto Talag and the other two locations (Alto Pano (B) and Alto Tena (C)) (Figure 3). These results may be related to soil properties: Alto Pano (B) is the richest in organic matter, nitrogen, and phosphorus; C contains moderate amounts of these nutrients, while A exhibits the lowest levels [45]. This suggests that nutrient availability may influence the accumulation and composition of secondary metabolites, reinforcing or modulating the plant’s defensive and protective strategies. Indeed, the availability and composition of mineral nutrients have a profound effect on the type and amount of primary and secondary metabolites produced [46].

In this sense, the metabolomic clustering revealed two distinct groups: one comprising B and C samples, and another defined by A (Figure 3 and Figure 4). In the B–C cluster, the profiles were enriched in phenols and flavonoids (Figure 3 and Figure 4), which strongly correlated with antioxidant parameters (ABTS, DPPH, TPC), indicating higher antioxidant potential (Figure 5 and Figure 6). In contrast, C was characterized by higher levels of alcohols, aldehydes, and hydrocarbons, compounds more related to structural and defensive roles rather than antioxidant activity (Figure 3 and Figure 4) [47,48,49,50]. This separation highlights a clear divergence in the chemical ecology of the populations.

Conversely, all samples displayed distinct chemical profiles between young (0) and old leaves (2). Leaf age can significantly influence the accumulation of secondary metabolites in plants. While younger leaves often prioritize the rapid synthesis of defensive compounds against herbivores and pathogens, mature leaves tend to accumulate higher levels of certain antioxidant metabolites [51,52,53,54]. Compounds such as phenolics and flavonoids are more abundant in older tissues, enhancing their protective roles against oxidative stress [51,52,53,54]. This increase reflects the adaptive strategy of plants to maintain cellular integrity during aging. Therefore, leaf maturity is strongly associated with elevated antioxidant potential; however, this pattern may vary depending on the specific physiological needs of the plant [55,56].

The volatile profile was consistent with the bioactivity assays. Young leaves of Alto Pano (B0) exhibited the strongest α-glucosidase inhibition, along with elevated TFC and high DPPH activity. These assays also showed strong Pearson correlations (Figure 5), supporting the consistency of the observed trends. The volatile composition of B0 was enriched in phenol, phenyl ethyl alcohol, senecioic acid, isopropyl tetradecyl ether, vanillin, syringol, 4-propenylsyringol, and methyl cinnamate. All of these compounds displayed strong correlations with α-glucosidase inhibition, as well as with ABTS activity, and to a lesser extent with DPPH (Figure 6A). In the biplot analysis, they clustered in the positive PC1 with the assays of α-glucosidase, ABTS, and TFC, confirming their positive associations and reinforcing the significant correlation with DPPH activity (Figure 6B). However, B0 includes 4′,6,7-trimethoxyflavonol, which showed a moderate correlation with α-glucosidase inhibition and a significant correlation with DPPH activity (Figure 6A). In the biplot analysis, this compound was located on the negative axis of PC1, corroborating its strong association with DPPH activity and moderate contribution to α-glucosidase inhibition and (Figure 6B).

Among these compounds, phenol, syringol, 4-propenylsyringol, methyl cinnamate, and vanillin possess structural features that enable them to stabilize free radicals through hydrogen donation, supporting their reported antioxidant activity [57]. For instance, syringol, identified from *Rhizophora apiculata* distillation, has demonstrated its antioxidant activity for DPPH and ABTS [58]. Similarly, methyl cinnamate has been reported as one of the key contributors to the antioxidant activity of *Clinopodium brownei* essential oil [59], and has also been described as a highly active antioxidant compound with efficacy comparable to that of vitamin E [60]. In addition, the potential α-glucosidase inhibitory activity of methyl cinnamate may be attributed to the presence of its carbonyl group, which can form hydrogen bonds with residues in the enzyme’s active site, along with possible hydrophobic interactions facilitated by its aromatic ring [57]. While methyl cinnamate itself has not been extensively studied in isolation for this activity, other cinnamic acid derivatives have shown notable α-glucosidase inhibitory effects, highlighting their potential therapeutic relevance [61,62]. In addition, vanillin is particularly noteworthy, as it has been previously identified in other *Ilex* species, such as *Ilex pubescens*, and reported as a potential α-glucosidase inhibitor through both in vitro assays and in silico molecular modeling [63]. Evidence suggests that vanillin can reduce enzyme activity through two complementary mechanisms: (i) direct binding to the active site, thereby blocking substrate interaction, and (ii) induction of conformational changes that reduce the thermal stability of the enzyme, ultimately altering its three-dimensional structure [63].

In contrast, B2 exhibited higher TPC and stronger ABTS activity, which were positively correlated across assays. The volatile composition was characterized by a higher relative abundance of alcohols and fatty acid derivatives with reported antioxidant capacity, including tolualdehyde, isopropyl tetradecyl ether, 1-hexadecanol, and 2(5H)-furanone (Figure 4), all of which showed strong Pearson correlations (Figure 6A) and clustered on the positive axis of PC1 in the biplot analysis near ABTS and TPC (Figure 6B). Additionally, isophytol and isopentyl valerate displayed strong correlations with DPPH activity (Figure 6A) and clustered on the negative axis of PC1, reinforcing their association with DPPH (Figure 6B).

Molecular docking analysis provided mechanistic evidence at the molecular level that supports the reported bioactivity of *I. guayusa* extracts (Table 5). The free binding energy values (ΔG) and the nature of the interactions identified allow us to establish a clear structure-activity relationship for the inhibition of α-glucosidase and catalase enzymes, which are related to antidiabetic and antioxidant properties, respectively. The metabolite with the highest in silico binding affinity was 4′,6,7-trimethoxyflavonol, with energies of −8.3 kcal/mol (α-glucosidase) and -9.4 kcal/mol (catalase). Its high affinity is mediated by multivalent interactions with key catalytic residues. In α-glucosidase (3A4A), it formed conventional hydrogen bonds with Arg315 and Asp242, and a T-type π–π stacking with Tyr158. In catalase (2CAG), it established hydrogen bonds with Arg51 and Arg344, and a π–π stacking with Tyr337. The methylation pattern of the flavonoid increases its lipophilic character, which favors better coupling in the hydrophobic cavities of the active sites. This finding is consistent with the literature, where flavonoid methylation is associated with an increase in affinity for enzyme targets such as α-glucosidase, by optimizing van der Waals interactions and reducing the entropic penalty upon binding [64,65].

A positive correlation was observed between predicted affinity and the complexity of non-covalent interactions. Metabolites with aromatic substituents (methoxy, propenyl), such as 4-propenylsyringol (7), vanillin (5), and syringol (8), generated networks of hydrogen bonds, π–π stacking, and simultaneous hydrophobic contacts, anchoring themselves stably in the catalytic pockets. In contrast, smaller ligands without these structural motifs, such as phenol (6) or senecioic acid (2), showed weaker and more peripheral bonds, with bond energies ≥ −5.6 kcal/mol.

The docking results complement the previous statistical correlation analyses. Compounds that showed a strong correlation with antioxidant and anti-α-glucosidase activity (e.g., vanillin, syringol) also exhibited significant affinities (ΔG ≤ −5.5 kcal/mol) and specific interactions with critical residues. For example, vanillin overcame an unfavorable donor-donor interaction with Asn317 (in 3A4A) through π–π stacking with Phe314 and a π-cation interaction with His423. This concordance suggests that these compounds are direct contributors to the bioactivity of the extract.

The apparent discrepancy between the docking results and the statistical correlation analyses can be explained through complementary perspectives. The high predicted affinity for 4′,6,7-trimethoxyflavonol, despite its moderate in vitro correlation, suggests that its individual inhibitory activity could be masked in the crude extract assay. This masking could be due to a relatively low concentration within the complex mixture of metabolites or, more interestingly, to its mechanism of action being enhanced by synergistic effects with other compounds present, which are not captured in linear correlation assays or individual docking experiments.

Conversely, metabolites that showed a strong statistical correlation with biological activity but low in silico binding affinity probably do not act as direct inhibitors of the modeled enzyme targets. Instead, their strong association suggests that they could function as excellent chemical markers for identifying active extracts, where their presence indicates the co-occurrence of other more potent inhibitory compounds. Alternatively, these metabolites could exert their biological activity through complementary mechanisms not evaluated in this model, such as modulation of cellular signaling pathways, interaction with other protein targets, or enhancement of the bioavailability of other active ingredients [66,67]. In catalase, the binding of the highest-affinity ligands involved residues essential for H_2_O_2_ catalysis, such as Arg91, Arg344, and Tyr337 [68,69]. The interaction with Tyr337, a conserved residue, through π–π stacking—observed in six of the nine metabolites evaluated—suggests a potential mechanism of competitive or allosteric inhibition, consistent with crystallographic studies of catalase-ligand complexes [70].

In this study, we identified 4′,6,7-trimethoxyflavonol, vanillin, 4-propenylsyringol, and syringol as the main contributors to the inhibitory activity toward α-glucosidase and catalase, providing a rational basis for the antidiabetic and antioxidant effect of *I. guayusa* at the molecular level. The in vitro validation of these compounds and the analysis of the stability of the complexes using molecular dynamics are the next steps in this research. Additionally, the ADME profile indicates that most *I. guayusa* metabolites possess suitable characteristics for development as oral α-glucosidase inhibitors (Table 6). These compounds demonstrate high intestinal absorption (GIA > 77%) and favorable Caco-2 permeability (>1.0 × 10^−6^ cm/s), supporting their oral bioavailability, which is crucial for antidiabetic drugs acting in the digestive tract [71]. All compounds comply with Lipinski’s Rules, reinforcing their pharmaceutical potential, though the elevated molecular weights of Comp3 and Comp9 may limit their lead-likeness [72]. Low penetration into the SNS is advantageous for minimizing central adverse effects, such as sedation or neurotoxicity [73]. However, the inhibition of CYP450 by Comp9 (CYP1A2, CYP2C19, CYP3A4) raises concerns about potential drug–drug interactions with medications like warfarin and antiretrovirals [74]. The moderate solubility of Comp3 and Comp9 could affect their oral formulation, suggesting the need for strategies such as nanocarrier technology [75]. Comp1, Comp2, Comp5, and Comp8 emerge as particularly promising candidates due to their optimal combination of high absorption, good solubility, and absence of CYP450 interactions. In contrast, Comp9, despite its high absorption, requires safety evaluation due to its multisystemic inhibition of CYP450 and potential renal accumulation (OCT2 and P-gp substrate). Additionally, results reveal that most compounds lack significant toxicity alerts, though important exceptions exist (Table 7). Comp4 presents substantial safety concerns due to its high carcinogenic probability (Pb = 0.84) and low acute toxicity threshold (LD_50_ = 100 mg/kg), making it unsuitable for therapeutic use without structural modification. Comp7 shows immunotoxic and mutagenic activity despite its moderate LD_50_ (1560 mg/kg), potentially limiting its clinical application. In contrast, Comp2 and Comp3 demonstrate particularly favorable safety profiles, exhibiting no specific toxicity alerts along with high LD_50_ values (2450 and 5000 mg/kg, respectively), indicating wide therapeutic windows. While Comp9 shows low acute toxicity (LD_50_ > 2000 mg/kg), its immunotoxic potential suggests that any therapeutic use would require careful evaluation, potentially restricting it to non-chronic treatments or mandating additional immunological safety assessments. These findings highlight the need for compound-specific risk-benefit analyses when considering clinical development of *I. guayusa* metabolites.

Then, in general, most of the analyzed compounds did not represent a relevant risk which was similar to results shown in the in vitro evaluation of extracts against red blood cells, demonstrating less than 1% of hemolytic activity in all samples (Figure 7). It is meaningful that so many authors mention less than 10% as the acceptable value to consider a plant extract for pharmacological aims [76,77,78,79,80,81,82]. Despite the in vitro hemolytic assay do not cover the broad predicted results of in silico analysis, it has demonstrated to be mainly used as it is rapid, reproducible, and inexpensive compared to some tests such as cell culture [76,77,78,79,80,81,82,83]. Although, red blood cells have been reported as a good option for the preliminary evaluation of natural products in vitro toxicity thinking about human use [83]. Moreover, it lets us evaluate the extract as the complex sample that it is, giving as a first glance its toxicity considering the molecules acting in synergy. So, it not only supports and complements the ADME results but highlights the potential clinical applications of these extracts. Safety profile obtained is pertinent as plant extracts have been proposed as alternative or complementary approaches for the management of various diseases [78]. It helped us to predict and better understand the toxicity of samples, which constitutes a crucial first step, as it can reveal potential membrane-disruptive effects of a natural extract that could limit its therapeutic applicability [83].The results suggest that *Ilex guayusa* extracts have promising applications beyond their well-known energizing effects. The biological activities observed in these complex extracts are unlikely to be attributed to a single compound but are rather the result of synergistic interactions among phenolic and flavonoid compounds, as demonstrated in other species such as *Astragalus membranaceus* and *Paeonia lactiflora* [84,85]. This synergy can enhance overall bioactivity beyond the sum of individual effects. This study underscores the importance of evaluating whole extracts, particularly in the *Ilex* genus where research on metabolite synergy remains limited [86]. In line with this, our future research plans include the fractionation of extracts into different chemical classes, followed by reconstitution assays, to evaluate the contribution of specific fractions and their combinations. Such strategies will provide concrete evidence of synergistic effects and deepen the understanding of the mechanisms behind the bioactivity of Amazonian plant resources.

## 4. Materials and Methods

### 4.1. Plant Collection

Sampling sites were located in the Napo province, distributed in Alto Talag (A) (−1.0832° S, −77.8902° W, 504 masl), Alto Pano (B) (−1.0022° S, −77.8933° W, 665 masl) and Alto Tena (C) (−0.9393° S, −77.8753° W, 660 masl). At each site, under shade conditions (0 to 200 μmol m^−2^·s^−1^) guayusa leaves were collected considering early (4–6 years) and adult (8–10 years) ages. Tree age was used to classify samples into “early” (4–6 years) and “adult” (8–10 years). Age was determined based on information provided by local farmers and corroborated by measuring the approximate trunk diameter at 1 m above the ground (mean diameter ~4 cm for early trees and ~8 cm for late trees). To obtain representative samples of each factor combination, leaves were collected from multiple branches of each tree. Sampling was conducted at a single time point for all trees to minimize potential bias due to leaf growth or replacement. Furthermore, leaf collection followed the traditional harvesting practices of local farmers to preserve the cultural representativeness of the sampling. Samples were stored in paper bags inside coolers at 4 °C and transported to the laboratory for further processing. Samples were collected under the Genetic Resources Access Permit: MAATE-CMARG-2023-0017.

### 4.2. Extracts Preparation

Leaves were rinsed with ultrapure water and dried at 35 °C for three days, then ground into a fine powder. Extracts were prepared by maceration, at room temperature, for five days using a 20% (w/v) ratio and 70% ethanol as the extraction solvent. To enhance metabolite recovery, the mixtures were sonicated for 20 min daily. After extraction, the solutions were filtered and concentrated by rotary evaporation at 100 rpm. The resulting concentrates were subsequently lyophilized under 120 mTorr at −80 °C.

### 4.3. Total Phenol Content

The phenolic content was analyzed by the Folin–Ciocalteu (Supelco^®^) colorimetric method [87]. 1 mg/mL of each sample was mixed with 860 μL of water and 40 μL of Folin–Ciocalteu in light-absent conditions and incubated for 5 min at room temperature. Following this, 100 μL of a 7% Na_2_CO_3_ solution was added, and the mixture was left to stand for 60 min. The absorbance of the resulting mixture was measured at 750 nm using a Shimadzu UV-1280 spectrophotometer (Shimadzu Corporation, Kyoto, Japan). A calibration curve of gallic acid (50 to 160 μg/mL) was used as a reference standard. The total phenolic content of the extracts was expressed as μg of gallic acid equivalent per mg of extract.

### 4.4. Total Flavonoid Content

The total flavonoid content was determined using the aluminum trichloride (AlCl_3_) method [88]. 1 mL of each sample (1 mg/mL) was mixed with 1 mL of 2% AlCl_3_ and incubated at room temperature for 10 min. The absorbance was measured at 438 nm using a Shimadzu UV-1280 spectrophotometer (Shimadzu Corporation, Kyoto, Japan). A calibration curve was prepared using quercetin as a standard within the range of 1–10 μg/mL. The results were expressed as μg of quercetin per mg of extract.

### 4.5. Antioxidant Activity

To evaluate antioxidant activity, DPPH (2,2-diphenyl-1-picrylhydrazyl radical) and ABTS (2,2′-azino-bis(3-ethylbenzothiazoline-6-sulfonic acid) radical) assays were used.

#### 4.5.1. DPPH Radical Scavenging Assay

The assay of DPPH was carried out according to the method outlined by [89], with slight modifications. In quartz cuvettes, 2.0 mL of sample (or Trolox standard) was mixed with 2.0 mL of ethanolic DPPH (0.10 mM), incubated for 30 min at room temperature in the dark, and the absorbance at 517 nm was recorded on a Shimadzu UV-3600 Plus spectrophotometer (Shimadzu Corporation, Kyoto, Japan). Percent inhibition was calculated using the following Equation (1):

(1) $$DPPH\;Radical\;Scavenging\;Inhibition\;\left(\%\right)=\frac{Ac-As}{Ac}*100$$

where Ac is the absorbance of the DPPH control and As is the absorbance of the sample.

Measurements were made in triplicate at 5–6 concentrations spanning ~20–80% inhibition. IC_50_ values were obtained by linear interpolation between the two concentrations bracketing 50% inhibition, and antioxidant capacity was expressed as TEAC (IC_50__Trolox/IC_50__sample), with Trolox analyzed on the same day.

#### 4.5.2. ABTS Radical Scavenging Assay

The ABTS assay was estimated using the method of [90], with some modifications. Trolox (6-hydroxy-2,5,7,8-tetramethylchroman-2-carboxylic acid) was used as a standard. Firstly, the ABTS+ radical cation was generated by incubating 7 mM of ABTS aqueous solution with 2.45 mM potassium persulfate in a light-absent condition. The working solution was obtained by diluting the ABTS+ solution with ethanol until it reached an absorbance of 0.70 ± 0.02 at 734 nm, evaluated using a spectrophotometer (Shimadzu 3600 Plus, Shimadzu Corporation, Kyoto, Japan). Then, 150 μL of samples or standard solutions prepared at varying concentrations using ethanol as solvent, was added to 2850 μL of the ABTS+ solution. The absorbance was measured at 734 nm after 6 min incubation at room temperature.

The Trolox standard solution was used to construct the calibration curves, and the results are expressed as mg Trolox equivalents/grams of dry weight; the results are an average of three independent measurements. The ABTS activity was expressed as a percentage of inhibition and calculated using the following Equation (2):

(2) $$ABTS\;Radical\;Scavenging\;Inhibition\;\left(\%\right)=\frac{Ac-As}{Ac}*100$$

where Ac is the absorbance of the ABTS control and As is the absorbance of the sample, measured at the same reaction time.

### 4.6. Antidiabetic Activity

#### 4.6.1. α-Amylase Inhibition

α-amylase inhibition was evaluated following the method reported in the literature [91,92]. Sodium phosphate buffer (0.02 M) (pH 6.9, containing 0.06 M NaCl) was used as the main solution. 25 μL of the sample with 25 μL of porcine pancreatic α-amylase (0.5 mg/mL) was incubated at 37 °C for 10 min. Subsequently, 1% w/v starch solution was added and incubated at 37 °C for 15 min. The reaction was stopped by adding a dinitrosalicylic acid (DNS) reagent, followed by heating at 100 °C. Finally, absorbance was measured at 540 nm. Acarbose was used as a reference standard, and the percentage of inhibition was calculated using the following Equation (3):

(3) $$Enzymatic\;inhibition\;\left(\%\right)=\frac{Ang-As}{Ang}*100$$

where Ang is the absorbance of the negative control and As is the absorbance of the sample.

#### 4.6.2. α-Glucosidase Inhibition

α-glucosidase inhibition was evaluated through the inhibition activity of the α-glucosidase enzyme assay. Sodium phosphate buffer (0.02 M) (pH 6.8), 0.2 U of α-Glucosidase from Saccharomyces cerevisiae enzyme, and samples were initially incubated at 37 °C for 10 min. Then, p-Nitrophenyl α-D-glucopyranoside (pNPG) was added as the substrate and the assay was incubated again at 37 °C for 30 min. Finally, 0.2 M Sodium Carbonate (Na_2_CO_3_) was added as a stop solution. Enzyme activity was measured at 405 nm absorbance, using a microplate reader. Acarbose was considered as positive control. The percentage of enzyme inhibition was determined using the following Equation (4):

(4) $$Enzymatic\;inhibition\;\left(\%\right)=\frac{Ang-As}{Ang}*100$$

where Ang is the absorbance of the negative control and As is the absorbance of the sample.

### 4.7. Chemical Characterization by GC-MS

#### 4.7.1. Sample Preparation

Sample preparation was carried out by diluting 5 mg of each lyophilized extract in 1 mL of ethanol. The mixture was homogenized and centrifuged at 10,000 rpm for 10 min. The resulting supernatant was filtered through a 0.22 μm Whatman Uniflo syringe filter (Cytiva, Freiburg im Breisgau, Germany) and transferred into a vial.

#### 4.7.2. Untargeted Metabolomic Approach and Data Analysis

The chemical profile of the samples was characterized using a GC-MS system consisting of an AOC-6000 autosampler (Shimadzu Corporation, Kyoto, Japan) coupled to a GCMS-QP2020 NX quadrupole mass spectrometer (Shimadzu Corporation, Kyoto, Japan) operating in electron ionization (EI) mode. Chromatographic separation was achieved on an Rtx-5MS capillary column (30 m × 0.25 mm i.d., 0.25 μm film thickness) coated with a stationary phase of 5% diphenyl and 95% dimethylpolysiloxane. A 1.0 μL aliquot of the extract was injected, and the oven temperature was programmed to start at 70 °C, increase at 6 °C/min, and reach a final temperature of 300 °C, held for 10 min, totaling 47.45 min of runtime. Helium (ultrapure grade) was used as the carrier gas at a constant flow rate of 1 mL/min. The injector and transfer line were maintained at 200 °C and 220 °C, respectively, while the ion source operated at 200 °C with 70 eV ionization energy. Mass spectra were acquired over an *m*/*z* range of 50–500 Da. For metabolite feature extraction, raw Shimadzu data (.qdf format) were converted to .mzML using ProteoWizard v3.0.21229. The files were processed in MS-DIAL v4.9.221218 (http://prime.psc.riken.jp/, accessed on 15 February 2025) and Mzmine 4.3.0 through a workflow comprising peak detection, alignment, gap filling, and blank subtraction, considering only features where the sample-to-blank intensity ratio exceeded 7. The resulting feature table (*.txt) was imported into the notame package in R for preprocessing steps including normalization and signal drift correction. Finally, the processed dataset was uploaded to MetaboAnalyst 6.0 for multivariate statistical analysis. The complete statistical analysis has been uploaded to the Github repository (https://github.com/thomasgarzon-cpu/Ilex-Guayusa-antidiabetic-and-antioxidant, accessed on 10 August 2025).

### 4.8. Statistical Analysis

All analyses (ABTS, DPPH, TPC, TFC, α-glucosidase, and GC–MS) were performed in triplicate, and results are presented as mean ± standard deviation. Significant differences among samples were evaluated by analysis of variance (Type I ANOVA) followed by Tukey’s HSD post hoc test and are indicated with different lowercase letters at *p* < 0.05. Correlations between the assays and metabolite abundances were examined using Pearson’s correlation coefficient (*r*). The statistical significance of each correlation was assessed with a two-tailed test, and *p*-values were adjusted for multiple comparisons using the Benjamini–Hochberg false discovery rate (FDR) method. All statistical analyses were conducted in R (version 4.4.0), also the raw data, analysis scripts, and exact *p*-values are available in our GitHub repository (https://github.com/SaborCanela/Unlocking-the-Ilex-guayusa-Potential-Volatile-Composition-Antioxidant-Antidiabetic, accessed on 10 March 2025).

### 4.9. Hemolytic Activity

The toxicity on red blood cells was assessed following [93], methodology, with slight modifications. 200 μL of a 4% (*v/v*) suspension of red blood cells with PBS 1X as solvent was mixed with 200 μL of several extract concentrations (400, 200, 160, 120, 80, 40, 5 μg/mL). Then, it was incubated at 37 °C for 120 min. PBS 1X and 2% (*v/v*) Triton X-100 (Sigma-Aldrich, St. Louis, MO, USA) were considered as negative and positive controls, respectively. Five replicates per extract concentration and controls were performed. After incubation, samples were centrifuged at 1000× *g* for 5 min, and 200 μL of supernatant was transferred to a 96-well sterile plate. Lysis of red blood cells was measured at λ 570 nm in a Glomax plate reader (Promega, Madison, WI, USA). The percentage of hemolysis was calculated as the following Equation (5):

(5) $$Hemolysis\;\left(\%\right)=\frac{As-Ang}{Apc-Ang}*100$$

where As is the absorbance of the sample, Ang the absorbance of the negative control, and Apc the absorbance of the positive control.

### 4.10. In Silico Molecular Docking Coupled with ADME Prediction

#### 4.10.1. Molecular Docking Analysis

The crystal structure of the target proteins was retrieved from RCSB Protein Data Bank (RCSB PDB; https://www.rcsb.org/, accessed on 10 April 2025). These included isomaltase from *Saccharomyces cerevisiae* (PDB ID: 3A4A) with a resolution of 1.6 Å and catalase (PDB ID: 2CAG) with a resolution of 2.7 Å. Using AutoDock Tools 1.5.6, heteroatoms and water molecules were removed, and polar hydrogens and Kollman charges were added to the proteins [68,94,95]. Ligand binding sites were identified using PrankWeb (https://prankweb.cz/, accessed on 20 April 2025), a machine learning-based tool that predicts binding pockets from protein structures [96]. The server analyzed the 3D structure of 3A4A and 2CAG, identifying key amino acids at the binding sites. The ligands, characterized by UPLC-MS/MS (Waters, MA, USA) in extracts from *Ilex guayusa*, were obtained from PubChem (https://pubchem.ncbi.nlm.nih.gov/, accessed on 21 April 2025) and optimized with the MMFF94 force field in Avogadro v1.2.0 [97]. The files were converted into PDB format with OpenBabel v2.4.1 [98]. Molecular docking was performed with AutoDock Vina v1.2 [99] using a grid box centered at (21.519, −7.702, 23.554) for protein 3A4A and at (59.5647, 14.8787, 16.3862) for 2CAG. The grid dimensions were set to 30 × 30 × 30 and 60 × 60 × 60 points for 3A4A and 2CAG, respectively. The protein was kept rigid, while the ligands retained bond rotations. For each compound, 50 independent simulations were run, recording the average free binding energy (ΔG) of the 20 best poses. The interactions were visualized in 2D/3D with Discovery Studio v24.1.0 [100].

#### 4.10.2. In Silico ADMET Profiles of Relevant Molecular Docking Compounds

The pharmacokinetic and toxicological profiles of the compounds were evaluated in silico using their SMILES structures obtained from PubChem. For the analysis of ADME properties, SwissADME (http://www.swissadme.ch/, accessed on 21 April 2025) was used, which determined key parameters such as lipophilicity (LogP), aqueous solubility (LogS), intestinal absorption (AIE), blood–brain barrier (BBB) permeability, and compliance with Lipinski’s rule (molecular weight ≤ 500 Da, LogP ≤5, ≤5 hydrogen bond donors, and ≤10 hydrogen bond acceptors) [101]. The tool also generated radar charts to evaluate bioavailability, considering polarity, size, solubility, and molecular flexibility. To complete the pharmacokinetic profile, pkCSM predicted interactions with CYP450 enzymes, distribution parameters (volume of distribution, total clearance), and excretion (specificity by OCT2 transporters) [102]. Finally, the toxicological evaluation was performed with ProTox 3. 0 (https://tox.charite.de/, accessed on 21 April 2025), which classified toxicity according to the Globally Harmonized System (GHS), reporting LD50 values [mg/kg] for oral exposure, as well as predictions of hepatotoxicity, carcinogenicity, mutagenicity, and hERG channel inhibition, complemented by dermal toxicity and mutagenicity analyses (AMES test) [103].

## 5. Conclusions

The analysis of the chemical and biological activities of guayusa extracts revealed that factors such as leaf age and growth location significantly influence their chemical composition, which in turn affects their biological activity. Belonging age, younger leaves supported the known pattern of compounds more related to plant defense function, while older leaves, containing metabolites more related to antioxidant activity, looking for fight against the oxidative stress of maturity. Also, molecular docking and correlation statistical analysis demonstrated useful techniques for better understanding and discovery of more related compounds for every biological activity, such as antioxidant and α-glucosidase inhibition, both of which were notably in guayusa. It is remarkable considering this species also revealed low toxicity, highlighting its potential pharmacological applications.

While the data presented are valuable, it is important to acknowledge that chemical composition may vary depending on environmental and physiological factors. Future studies could benefit from investigating potential synergistic interactions among bioactive compounds to better elucidate the mechanisms of action in these complex extracts.

Overall, this study provides a foundation for understanding the therapeutic potential of guayusa, supported not only by in vitro assays but also by predictive ADMET analyses. In addition to its academic relevance, these findings may have practical implications for agriculture and entrepreneurship by promoting the valorization of biological resources with economic and cultural value.

## Figures and Tables

**Table 1:** Quantitative phytochemical analysis of hydroethanolic extract of *I. guayusa*.

Sample	Total Phenolic Content (TPC) (μg GAE/mg Extract) *	Total Flavonoids Content (TFC) (μg QE/mg Extract) *
A0	39.59 ± 0.33 ^d^	6.19 ± 0.01 ^b^
A2	34.58 ± 0.25 ^f^	5.95 ± 0.03 ^bc^
B0	56.84 ± 0.20 ^b^	6.77 ± 0.07 ^a^
B2	77.91 ± 0.16 ^a^	6.11 ± 0.18 ^bc^
C0	54.44 ± 0.25 ^c^	5.85 ± 0.58 ^c^
C2	36.58 ± 0.11 ^e^	4.27 ± 0.12 ^d^

* Values are expressed as mean ± SD (n = 3). Superscript lowercase letters (“a–f”) within the same column indicate statistically significant among the samples from different locations. Sampling sites: A = Alto Talag, B = Alto Pano, C = Alto Tena. Leaf age: 0 = young leaves, 2 = old leaves. Means followed by different letters indicate significant differences (*p* < 0.05, Tukey’s HSD test).

**Table 2:** Antioxidant activity of hydroethanolic extract of *I. guayusa*.

Sample	TEAC (mg/gdw) *	
	ABTS	DPPH
A0	0.3184 ± 0.0004 ^Ad^	0.3033 ± 0.0313 ^Aa^
A2	0.2906 ± 0.0081 ^Ae^	0.0968 ± 0.0026 ^Bc^
B0	0.3577 ± 0.0028 ^Ab^	0.2807 ± 0.0236 ^Ba^
B2	0.3885 ± 0.0459 ^Aa^	0.2264 ± 0.0049 ^Bb^
C0	0.3377 ± 0.0067 ^Ac^	0.0551 ± 0.0089 ^Bd^
C2	0.3077 ± 0.0022 ^Ad^	0.1850 ± 0.0047 ^Bc^

* Values are expressed as mean ± SD (n = 3). Sampling sites: A = Alto Talag, B = Alto Pano, C = Alto Tena. Leaf age: 0 = young leaves, 2 = old leaves. Means followed by different lowercase letters within the same column indicate significant differences among samples (*p* < 0.05, Tukey’s HSD test). Superscript lowercase letters (“a–e”) within the same column indicate statistically significant among the samples from different locations and uppercase letters (“A, B”) within the same row indicate significant differences between antioxidant assays (ABTS vs. DPPH) for each sample.

**Table 3:** IC_50_ α-glucosidase inhibition of the hydroethanolic extract of *I. guayusa*.

Sample	IC_50_ (µg/mL)
Acarbose	48.79 ± 0.80
A0	251.31 ± 4.15 ^a^
A2	158.92 ± 11.30 ^b^
B0	68.05 ± 5.69 ^c^
B2	172.85 ± 8.06 ^b^
C0	286.77 ± 11.20 ^d^
C2	283.56 ± 6.32 ^d^

A: Alto Talag. B: Alto Pano. C: A Alto Tena. 0: Young leaves. 2: Old leaves. Means with different letters differ significantly (*p* < 0.05, Tukey HSD). Superscript lowercase letters (“a–d”) within the same column indicate statistically significant among the samples from different locations.

**Table 4:** Metabolites identified by GC-MS in *I. guayusa*.

IL *	RT (min) *	Score *	LTPRI Exp *	LTPRI Lit *	Identified Metabolite Name	Class	Biological Activity Reported
1	24.425	0.81	1862	1856.2	Caffeine	Imidazopyrimidines (xanthine derivative)	Central nervous system stimulant, improves concentration and alertness, diuretic effect, antioxidant, potential neuroprotective properties [30,31].
1	24.666	0.79	1984	1910	Theobromine	Xanthine (purine alkaloid)	Mild stimulant, vasodilator, diuretic, bronchodilator, cardiotonic, and antioxidant effects [32,33].
2	4.06	0.72	957.28	953.0	Senecioic acid	Fatty Acyls	Anti-inflammatory and antimicrobial activity in plant extracts [34].
2	5.127	0.81	1011.93	1004.1	Phenol	Phenol (hydroxybenzene)	Antiseptic, antimicrobial, corrosive at high concentrations, precursor in pharmaceutical synthesis [35].
2	8.06	0.90	1144.06	1141	Phenethyl alcohol (1-phenylethanol)	Aromatic alcohol	Antimicrobial, antifungal, used in perfumery for floral scent [36,37].
2	9.851	0.93	1216.61	1216	4-Vinylphenol	Vinyl phenol (functionalized phenol)	Antifungal activity [38,39,40].
2	10.474	0.86	1240.9	1237	Coumaran	Hydrogenated aromatic (indan derivative)	Some derivatives exhibit antioxidant and antimicrobial properties [41,42,43].
2	11.575	0.835	1283.2	1268.8	3-Methylcatechol	Methylated di-phenol	Antioxidant, inhibitor of oxidative enzymes, potential anticancer agent [44].
2	11.812	0.92	1293.08	1293	Indol	Aromatic heterocycle	Some derivatives have anticancer and antimicrobial activity [45,46].
2	11.818	0.75	1293.31	1287	Cinnamaldehyde	Aromatic aldehyde	Antimicrobial, anti-inflammatory, antioxidant, potential anticancer agent [47].
2	11.821	0.90	1293.41	1272	Hydroquinone	Di-phenol (1,4-dihydroxybenzene)	Skin depigmenting agent, antioxidant [48].
2	13.96	0.88	1277.25	1367	Syringol	Methoxylated phenol	Antioxidant, antimicrobial, lignin-derived compound [49,50,51].
2	15.029	0.82	1419.79	1398	Methyl cinnamate	Aromatic ester	Antimicrobial, pleasant aroma, used in perfumery and cosmetics [52].
2	15.151	0.74	1424.75	1420	Vanillin	Phenolic aldehyde	Antioxidant, antimicrobial, anti-inflammatory, natural flavoring agent.
3	18.131	0.79	1647.28	1532	Methyl laurate	Fatty acid ester	Antimicrobial and emollient activity, used in cosmetics and fragrances [53]).
3	3.223	0.77	913	2760	4′,6,7-Trimethoxyflavonol	Trimethoxyflavonol (polyphenol)	Antioxidant, anti-inflammatory, and anticancer activity [54].
3	3.976	0.82	952.87	924	2(5H)-Furanone	Furanone lactone	Antimicrobial, inhibitor of bacterial biofilm formation [55,56,57].
3	5.038	0.88	1007.71	900	Nonane	Alkane (C9)	No significant biological activity reported.
3	5.122	0.70	1011.73	1942	Isophytol	Acyclic terpene alcohol	Antioxidant properties, precursor to vitamins [58,59].
3	8.721	0.70	1171.15	1321.86	2,2,4,4,6,8,8-Heptamethylnonane	Highly branched alkane	No significant biological activity reported.
3	9.169	0.85	1189	2657	Catechol	Di-phenol (1,2-dihydroxybenzene)	Antioxidant, precursor in biosynthesis of other phenolic compounds, antimicrobial [60,61,62,63].
3	9.396	0.70	1198	1000	Hexadecane	Alkane (C16)	No significant biological activity reported.
3	10.371	0.86	1236	1089	Tolualdehyde	Aromatic aldehyde	No significant biological activity reported.
3	15.392	0.99	1434	897	4-Vinylphenol	Phenol	Antimicrobial phenolic compound affecting flavor and aroma of fermented foods [64,65].
3	18.226	0.70	1551	1500	Pentadecane	Alkane (C15)	No significant biological activity reported.
3	18.67	0.70	2078.47	2076	Isopropyl tetradecyl ether	Aliphatic ether	No significant biological activity reported.
3	21.26	0.70	1683.16	1152	Isopentyl valerate	Ester (carboxylic acid ester)	No significant biological activity reported.
3	22.265	0.70	1757.87	1642	4-Propenyl syringol	Functionalized phenol (propenylated)	Antioxidant and antimicrobial activities [66].
3	28.298	0.70	2228.07	1867	1-Hexadecanol	Aliphatic alcohol (C16 primary alcohol)	Emollient and mild antimicrobial properties [66,67].

* IL, identification level according to [68]; RT, retention time in minutes; Score indicates the degree of similarity between the experimental mass spectrum and the reference library spectrum; LTPRI Exp, experimental Van den Dool and Kratz retention index; LTPRI Lit, literature Van den Dool and Kratz retention index.

**Table 5:** Summary of binding affinities and key interactions of metabolites of *I. guayusa* with 3A4A, and 2CAG.

Cod	Ligand Name	3A4A		2CAG	
		Affinity (kcal mol^−1^)	Key Interactions	Affinity (kcal mol^−1^)	Key Interactions
9	4′,6,7-Trimethoxyflavonol	−8.3	Conventional H-bond: Arg315, Arg442. Carbon H-bond: Ser240, Asp242, Asp352, Leu313. π–π T-shaped: Tyr158. π-Alkyl: Arg315. Van der Waals: Leu313, Lys156, Phe314, Arg315, Glu411, Val216, Glu277, Asp352, Arg442, Phe159, Tyr158.	−9.4	Conventional H-bond: Arg51, Arg344. Unfavorable Donor–Donor: Arg344. π-Cation: Arg91. Carbon-Hydrogen Bond: Arg91. π–π Stacked: Tyr337. π–π T-shaped: Tyr337. Alkyl: Ala112, Val125, His54. π-Alkyl: Arg52, Ala340. Van der Waals: Tyr337, Asn127, Phe313.
7	4-Propenylsyringol	−5.5	Conventional H-bond: Asp242. Carbon H-bond: Ser241, Lys156. π–π T-shaped: Tyr158. Alkyl: Tyr158. π-Alkyl: Phe314. Van der Waals: Phe314, Leu313, Arg315, His280.	−7	Conventional H-bond: Arg333, His54. Carbon-Hydrogen Bond: Val125. π-Alkyl: His197. Alkyl: Phe140, Phe132, Val95. Van der Waals: Tyr337, Asn127, Gly126.
5	Vanillin	−5.9	Conventional H-bond: Lys156, Asn317. Carbon H-bond: Asp233. Unfavorable Donor–Donor: Asn317. π–π T-Shaped: Phe314. π–π Stacked: Phe314. π-Cation: His423. π-Alkyl: Ile419. Van der Waals: Phe314.	−6.4	Conventional H-bond: Arg333, Asn127, Ser196, Tyr337. Pi-Cation: His197. π–π Stacked: Phe132. π-Alkyl: His54. Van der Waals: Tyr337, Phe140, Gly126.
8	Syringol	−5.8	Conventional H-bond: Lys156. Carbon H-bond: Asn415, Asn235. π-Cation: His423. π–π Stacked: Phe314. Alkyl: Phe314. Van der Waals: Lys156, Leu313.	−6.3	Carbon-Hydrogen Bond: Val125. π-Cation: His197, Arg333. π–π Stacked: Phe132. Unfavorable Acceptor–Acceptor: Tyr337. Alkyl: His197, Arg333. π-Alkyl: Phe140. Van der Waals: Tyr337, Asn127, Gly126.
1	Phenylethyl alcohol	−5.6	Conventional H-bond: Asn415. Carbon H-bond: Asn415. π-Cation: His423. π–π Stacked: Phe314. π-Alkyl: Ile419. Van der Waals: Phe314, Lys156.	−6.1	Conventional H-bond: His197. π-Sulfur: Met329. π–π Stacked: Phe140. π-Alkyl: Arg333. Van der Waals: Tyr337, Asn127.
3	Methyl laurate	−5.1	Conventional H-bond: His351. Carbon H-bond: Asp215. Pi-Alkyl: Phe178, His112, Tyr158. Van der Waals: Arg442, Phe178, Tyr158, Glu277, Val216, Glu411, Gln279, Arg213.	−6.2	Conventional H-bond: Arg91. Carbon-Hydrogen Bond: Gly126. π-Alkyl: Ala112, Val125, His54. Alkyl: His341, Arg51, Tyr337, Ala340. Van der Waals: Tyr337, Phe313, Asn127.
4	Catechol	−5.6	Conventional H-bond: Glu277, His351, Glu277. π–π T-shaped: Tyr72. π -Cation: Arg442. Van der Waals: Glu411, Glu277, Asp352, Val216, His351, Arg315.	−5.6	Conventional H-bond: Arg91. π-Sigma: Ala112. π–π Stacked: His54. Pi-Alkyl: Val125. Van der Waals: Tyr337, Phe313.
6	Phenol	−5.3	Conventional H-bond: Arg213, Glu277, His351. π-Cation: Arg442. Van der Waals: Val216, Glu277, His351, Asp352.	−5.2	Pi-Sulfur: Met329. Pi–Pi Stacked: Phe140. π-Alkyl: Arg333. Van der Waals: Tyr337.
2	Senecioic acid	−5.2	Conventional H-bond: Arg442, His351, Asp352. Unfavorable Acceptor–Acceptor: Asp352. Alkyl: Val216. π-Alkyl: Phe178, His112. Van der Waals: Arg213, His351, Val216, Glu277, Asp352.	−5.2	Unfavorable Donor–Donor: Asn127. Alkyl: Met329, Leu278. π-Alkyl: Phe132, Phe140, His197. Van der Waals: Arg333, Phe140.

Van der Waals interactions are listed only if they involve critical residues.

**Table 6:** In silico ADMET analysis of nine compounds identified in the *I. guayusa* extracts.

PP	1	2	3	4	5	6	7	8	9
TPSA (Å2)	20.23	37.3	26.3	40.46	46.53	20.23	38.69	38.69	78.13
Consensus Log Po/w	1.64	0.89	4.1	0.97	1.2	1.41	2.28	1.32	2.73
MW (g/mol)	122.16	100.12	214.34	110.11	152.15	94.11	194.23	154.16	328.32
nRB	2	1	11	0	2	0	3	2	4
nOHA	1	2	2	2	3	1	3	3	6
nOHD	1	1	0	2	1	1	1	1	1
WLOGP	1.22	1.04	4.08	1.1	1.21	1.39	2.33	1.41	3.19
WS Log S (Ali)	V. Sol	V. Sol	M. Sol	V. Sol	V. Sol	V. Sol	Sol	V. Sol	M. Sol
GI absorption	High	High	High	High	High	High	High	High	High
DLLR	0	0	0	0	0	0	0	0	0
BS	0.55	0.85	0.55	0.55	0.55	0.55	0.55	0.55	0.55
Leadlikeness	1	1	3	1	1	1	1	1	0
Absorption									
WS (log mol/L)	−1.198	−0.314	−5.096	−0.441	−0.812	−0.723	−2.283	−0.979	−3.897
Caco2 (log Papp in 10^−6^ cm/s)	1.605	1.571	1.604	1.443	1.23	1.613	1.605	1.585	1.087
GIA. %	88.073	92.321	93.709	77.197	86.883	93.055	95.926	96.844	95.869
Skin Permeability log Kp (cm/s)	−1.83	−2.754	−1.844	−2.729	−2.752	−1.924	−2.438	−2.615	−2.712
P-gp substrate (Yes/No)	No	No	No	Yes	Yes	No	Yes	Yes	Yes
P-gp I inhibitor (Yes/No)	No	No	No	No	No	No	No	No	Yes
P-gp II inhibitor (Yes/No)	No	No	No	No	No	No	No	No	Yes
Distribution									
VDss (human) (log L/kg)	0.202	−0.833	0.256	−0.25	−0.357	0.131	−0.23	−0.331	−0.162
Fraction unbound (human)	0.434	0.667	0.23	0.491	0.464	0.533	0.355	0.457	0.14
BBB permeant (log BB)	−0.089	−0.248	0.674	−0.225	−0.182	−0.222	0.227	−0.139	−0.695
CNS permeability (log PS)	−1.857	−2.53	−1.897	−1.719	−1.828	−1.824	−1.661	−1.818	−2.309
Metabolism									
CYP2D6 substrate	No	No	No	No	No	No	No	No	No
CYP3A4 substrate	No	No	No	No	No	No	No	No	Yes
CYP1A2 inhibitor	Yes	No	No	No	Yes	No	Yes	Yes	Yes
CYP2C19 inhibitor	No	No	No	No	No	No	No	No	Yes
CYP2C9 inhibitor	No	No	No	No	No	No	No	No	No
CYP2D6 inhibitor	No	No	No	No	No	No	No	No	No
CYP3A4 inhibitor	No	No	No	No	No	No	No	No	Yes
Excretion									
Total renal clearance (log mL/min/kg)	0.325	0.894	1.724	0.173	0.595	0.208	0.242	0.207	0.662
Renal OCT2 substrate	No	No	No	No	No	No	No	No	Yes

PP: Physicochemical properties. TPSA: Topological Polar Surface Area, Consensus Log Po/w: average of all Log Po/w (octanol/water partition coefficient) values, MW: Molecular Weight, n-RB: Number of Rotatable Bonds, n-OHA: Number of hydrogen bond acceptors, n-OHD: Number of hydrogen bonds donors, WLogP: Logarithm of partition coefficient of compound between n-octanol and water (Wildman and Crippen), WS: Water solubility (V. Sol: Very soluble, M. Sol: Moderately soluble, Sol: Soluble), DLLR: Number of violations of Drug likeness Lipinski rule, BS: Bioavailability Score, Lead likeness violation violations. Caco-2: Colon adeno carcinoma permeability, GIA: Gastrointestinal absorption, P-gp: permeability glycoprotein, CNS: Central Nervous System permeability, PS: permeability surface area products, CYP: Cytochrome P450, VDss: Steady-state volume of distribution, BBB: Blood–Brain Barrier permeability, Renal OCT2 substrate: Organic cation transporter 2. Entry 1: Phenylethyl alcohol, 2: Senecioic acid, 3: Methyl laurate, 4: Catechol, 5: Vanillin, 6: Phenol, 7: 4-Propenylsyringol, 8: Syringol, 9: 4′,6,7-Trimethoxyflavonol.

**Table 7:** Toxicological properties of nine compounds from *I. guayusa* extracts.

Compounds	Hepatotoxicity		Carcinogenicity		Immunotoxicity		Mutagenicity		Cytotoxicity		Predicted LD_50_ (mg kg^−1^)	Toxicity Class
	Pr	Pb	Pr	Pb	Pr	Pb	Pr	Pb	Pr	Pb		
Phenylethyl alcohol	In	0.86	In	0.71	In	0.99	In	0.96	In	0.91	800	4
Senecioic acid	In	0.62	In	0.81	In	0.99	In	0.92	In	0.71	2450	5
Methyl laurate	In	0.58	In	0.55	In	0.99	In	0.98	In	0.73	5000	5
Catechol	In	0.82	Ac	0.84	In	0.99	In	0.9	In	0.87	100	3
Vanillin	In	0.52	In	0.6	In	0.55	In	0.98	In	0.94	1000	4
Phenol	In	0.8	In	0.77	In	0.99	In	0.99	In	0.91	270	3
4-Propenylsyringol	In	0.63	In	0.53	Ac	0.82	Ac	0.53	In	0.73	1560	4
Syringol	In	0.68	In	0.51	In	0.83	In	0.8	In	0.89	550	4
4′,6,7-Trimethoxyflavonol	In	0.69	In	0.57	Ac	0.78	In	0.7	In	0.99	5000	5

Pr: prediction; Pb: probability. In: Inactive; Ac: Active.

## Data Availability

The original data presented in the study are openly available in Github at https://github.com/SaborCanela/Unlocking-the-Ilex-guayusa-Potential-Volatile-Composition-Antioxidant-Antidiabetic, accessed on 10 August 2025.

## Supplementary Materials

The following supporting information can be downloaded at: https://www.mdpi.com/article/10.3390/molecules30193885/s1, Figure S1: Docking validation using the α-glucosidase inhibitor acarbose. (A) 3D pose of acarbose (ball and sticks) within the 3A4A active site. (B) 2D ligand-protein interaction diagram.

## Abbreviations

The following abbreviations are used in this manuscript:

GC-MS	Gas chromatography coupled to mass spectrometry
TPC	Total phenolic content
TFC	Total flavonoids content
ABTS	2,2′-azino-bis-(3-ethylbenzothiazoline-6-sulfonic) acid
DPPH	2,2-Diphenyl-1-picrylhydrazyl
LOQ	Limit of Quantification
SD	Standard Deviation
IL	Identification level
RT	Retention Time
PCA	Principal Component Analysis
HCA	Hierarchical cluster analysis
ADMET	Absorption, Distribution, Metabolism, Excretion, and Toxicity
CNS	Central Nervous System
GIA	Gastrointestinal absorption
IC_50_	Half inhibitory concentration
LD_50_	Half lethal dose
