# Supplementary material for: Unlocking the Ilex guayusa Potential: Volatile Composition, Antioxidant, Antidiabetic, and Hemolytic Activities, with In Silico Molecular Docking and ADMET Analysis of Hydroethanolic Extracts
Source: Molecules. 2025 Sep 25;30(19):3885. doi: 10.3390/molecules30193885 (PMC12526121; doi:10.3390/molecules30193885)
Supplement: Supplementary file 1 [file molecules-30-03885-s001.zip › molecules-3870489-supplementary.pdf]

Unlocking the *Ilex guayusa* Potential: Volatile Composition, Antioxidant, Antidiabetic, and Hemolytic Activities, with In Silico Molecular Docking and ADMET Analysis of Hydroethanolic Extracts

Nina Espinosa de los Monteros-Silva [1†,], Karla Martínez-Palacios [2†,] , Anggie Mikaela M. Jiménez[2], Melanie Ochoa-Ocampos[3], Thomas Garzón[2], Tamara Carrillo-Vásconez[2], Matteo Radice[4], Enith Vanessa Yanez[5], Julio Rea-Martínez[2], Zulay Niño-Ruiz[6], Karel Dieguez-Santana[2] and Noroska G.S. Mogollón [2*]

[1] Laboratorio de Biología Molecular y Bioquímica, Universidad Regional Amazónica Ikiam, Km7 Via Muyuna, Tena 150101, Ecuador; nina.espinosadelosmonteros@ikiam.edu.ec.

[2] Biomolecules Discovery Group, Universidad Regional Amazónica Ikiam, Km7 Via Muyuna, Tena 150101, Ecuador; karla.martinez@est.ikiam.edu.ec; anggie.medina@est.ikiam.edu.ec ; julio.rea@ikiam.edu.ec ; karel.dieguez@ikiam.edu.ec; gabriela.salazar@ikiam.edu.ec; thomas.garzon@ikiam.edu.ec; tamara.carrillo@ikiam.edu.ec

[3] Laboratorio de Productos Naturales, Universidad Regional Amazónica Ikiam, Km7 Via Muyuna, Tena 150101, Ecuador; melanie.ochoa@ikiam.edu.ec.

[4] Facultad de Ciencias de la Tierra, Universidad Estatal Amazónica, Puyo 160150, Ecuador; mradice@uea.edu.ec

[5] Grupo Traslacional en Plantas. Universidad Regional Amazónica Ikiam, Km7 Via Muyuna, Tena 150101 vanessa.yanez@ikiam.edu.ec.

[6] Biomass to resources Group, Universidad Regional Amazónica Ikiam, Km 7 Vía Muyuna, Tena, Napo 150101, Ecuador; zulay.nino@ikiam.edu.ec.

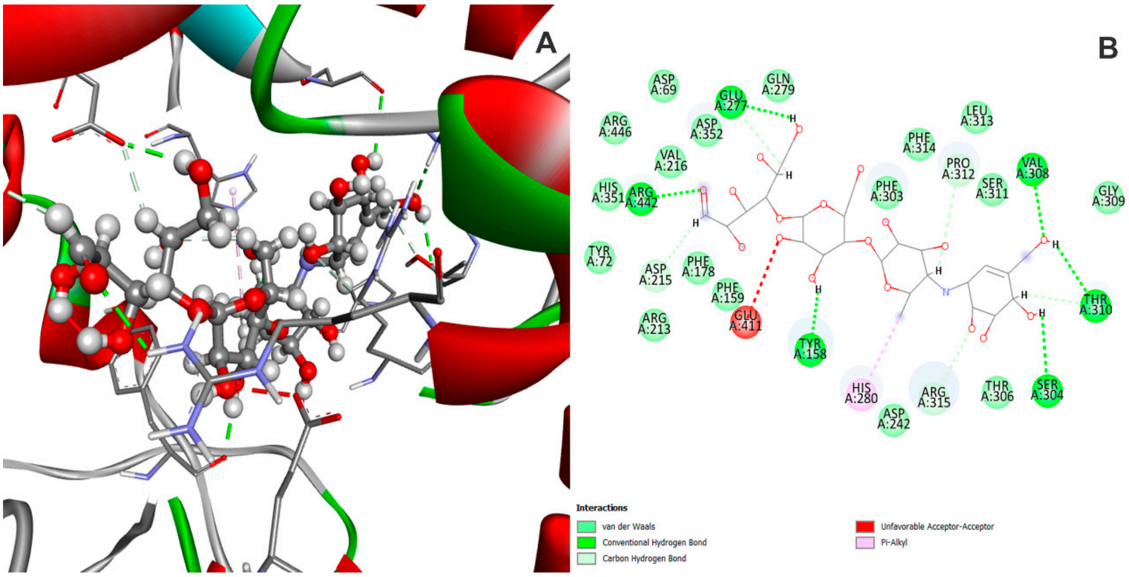

Figure S1. Docking validation using the α-glucosidase inhibitor acarbose. (A) 3D pose of acarbose (ball and sticks) within the 3A4A active site. (B) 2D ligand-protein interaction diagram.
